# Comparative Analysis of Clinical and Medication Information between Chronic Hepatitis B Patients with Damp Heat Syndrome and Spleen Deficiency Syndrome

**DOI:** 10.1155/2020/8846637

**Published:** 2020-12-28

**Authors:** Qiao-Hong Liu, Bin-Bin Zhang, Lin Xu, Xiao-Ping Shen, Ya-Mei Hai, Yi-Yang Hu, Yu Zhao

**Affiliations:** ^1^Key Laboratory of Liver and Kidney Diseases, Institute of Liver Diseases, Shuguang Hospital Affiliated to Shanghai University of Traditional Chinese Medicine, Shanghai 201203, China; ^2^Institute of Clinical Pharmacology, Shuguang Hospital Affiliated to Shanghai University of Traditional Chinese Medicine, Shanghai 201203, China; ^3^Shanghai Key Laboratory of Traditional Chinese Clinical Medicine, Shanghai 201203, China

## Abstract

Traditional Chinese medicine (TCM) has a long history in the treatment of chronic hepatitis B (CHB) based on the syndrome identification. Previous studies reported CHB patients with damp-heat (DH) syndrome accompanied with a severe liver function damage, but lacked the medication analysis. In this study, we analyzed 999 CHB patients with unidentified individual-level data from database to explore clinical features of two common syndromes of CHB patients based on the real world. Compared with the spleen deficiency (SD) syndrome, the CHB patients with DH syndrome had a significantly higher level of alanine aminotransferase (ALT) and aspartate aminotransferase (AST) (*P* < 0.05) but took more immunomodulators and hepatoprotective drugs (*P* < 0.1). Similarly, in the follow-up of 207 patients after 3 months, the improvement trend of ALT and AST of patients with sustained SD syndrome was significantly better than those whose TCM syndrome changed from SD to DH (*P* < 0.05). The logistic model indicated DH syndrome was a significant negative factor for reducing ALT level in CHB patients (OR = 4.854, *P*=0.032). This study suggests that CHB patients with DH syndrome have potentially more serious and sustained liver damage than the SD syndrome, which provides a reference for the personalized management of CHB patients from the perspective of TCM syndromes.

## 1. Introduction

Hepatitis B is caused by hepatitis B virus (HBV) infection. At present, the prevalence of hepatitis B surface antigen (HBsAg)-positive is about 4.9% worldwide [1]. China has a high incidence of hepatitis B. It is estimated that the prevalence of HBV infection in the general population of China is 5%–7.99% [2]. About 50% patients with HCC were complicated with HBV infection [3]; the Global Hepatitis Report 2017 shows that approximately 887 thousand people die of chronic hepatitis B- (CHB-) related complications worldwide in 2015, mainly due to liver cirrhosis and hepatocellular carcinoma. To prevent the prognosis of CHB, early diagnosis and effective treatment are very important.

The current treatment of CHB is mainly antiviral drugs, which can effectively block the replication of HBV-DNA and reduce the incidence of cirrhosis and HCC [4]. Traditional Chinese medicine (TCM) is widely used in China; it has the advantages of early treatment and combined intervention in the treatment of hepatitis B, which is reported to be beneficial for alleviating liver damage, reducing jaundice, regulating immunity [5], and inhibiting the development of liver fibrosis and HCC. TCM also was reported to lower the risk of death among CHB patients with contaminant liver cirrhosis and HCC [6–8]. Different from the strict indication screening of antiviral therapy, TCM can alleviate physical uncomfortable symptoms and improve the quality of life of CHB patients who are not suitable for antiviral therapy but have somatic discomfort symptoms such as pain in the liver area.

The characteristic of TCM is to provide personalized treatment based on the theory of Chinese medicine, in which the differentiation of TCM syndromes is the premise of personalized treatment. Since TCM and western medicine have common treatment subjects, whether there are clinical differences based on modern laboratory indicators in different syndromes has always been the focus of syndrome research. The damp-heat (DH) syndrome and spleen deficiency (SD) syndrome are the main subtypes of CHB according to the TCM syndrome differentiation [9, 10]. DH syndrome is reported to be accompanied by a higher liver injury in most of the literatures, but the liver function is easily interfered by medication in the real world. For example, some patients will quickly recover from liver damage after taking liver protection drugs. Existing studies compared the expression of liver function in patients with different syndromes but payed little attention to the usage of drugs [11–14], which cannot rule out the potential interference by the difference in medications. Besides, the TCM syndromes are not immutable; the dynamic longitudinal observation is necessary. By dynamically observing the changes in syndromes and combining with the medication, the clinical characteristics of different syndromes of CHB can be further clarified. Therefore, this study will analyze the clinical differences of two common TCM syndromes of CHB; we first explore the clinical indexes and medication differences between syndromes in a cross-sectional study and then confirm the clinical differences by longitudinal dynamic study analysis.

## 2. Methods

### 2.1. Study Design

**Figure 1:**
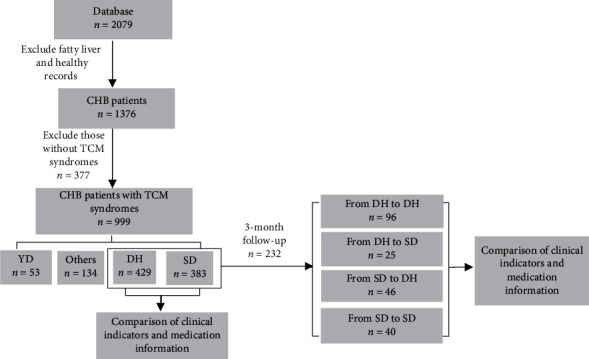
Study design.

All procedures followed were in accordance with the ethical standards of the responsible committee on human experimentation (institutional and national) and with the Helsinki Declaration of 1975, as revised in 2000 (5), and were approved by the IRB of Shuguang Hospital affiliated to Shanghai University of Traditional Chinese Medicine (Permit Number: 2012-206-22-01). Informed consent was obtained from all patients for being included in the study. This is a cross section study based on 2079 unidentified individual-level data from the database collected from 2008 to 2015, which consisted of two parts. The first data set contained 999 CHB patients with TCM syndrome differentiation records in the database; we described the distribution of syndrome types and compared the general information, clinical indicators, and drug usage between CHB patients with DH syndrome and SD syndrome. Among the patients in the first data set, 207 CHB patients were followed up after 3 months to form data set 2. We recorded the changes in the syndrome of these patients, compared the clinical indicators and medication information, and analyzed the related risk factors that affect the improvement of ALT (Figure 1).

### 2.2. Inclusion and Exclusion Criteria of Participants

Patients who met the diagnostic criteria of “chronic hepatitis B” [15] according to the “China Guidelines for Prevention and Treatment of Chronic Hepatitis B (2005)” [16], aged from 18 to 65, and signed informed consent were included. Patients who had viral hepatitis, chronic severe hepatitis, severe primary diseases, or mental diseases or were pregnant women or lactating women were excluded. The standard of the TCM syndrome differentiation was according to the “TCM Syndrome Differentiation Standard of Viral Hepatitis” [17] and “The Clinical Research Guide of New Drugs of Traditional Chinese Medicine” [18]; detailed syndrome differentiation standards are attached in Additional file 2.

### 2.3. Statistics

We used SPSS (version 26.0) for all statistical analyses. Shapiro–Wilk test was used to assess data normality. We evaluated the numerical variables that did not violate normality test and homogeneity test of variance by *T*-test, while the remaining numerical variables were compared using the Mann–Whitney *U*-test, and categorical data adopted the *χ*^2^ test. The normal distribution variables were expressed as mean ± standard deviation (*x* ± *s*); the nonnormal distribution variables were expressed using the median (median (1/4 quantile, 3/4 quantile)). The correlation analysis was estimated by the logistic regression. Simple logistic regression was conducted to estimate crude odds ratios (OR) and their 95% confidence intervals (95% CI) for all factors related to ALT differences. Multiple logistic regression was used to adjust potential confounding by age, gender, and other drug use. The differences were considered significant at *P* < 0.05.

## 3. Results

### 3.1. Comparison of Clinical Indexes and Medication of CHB Patients with DH Syndrome and SD Syndrome at Baseline

**Figure 2:**
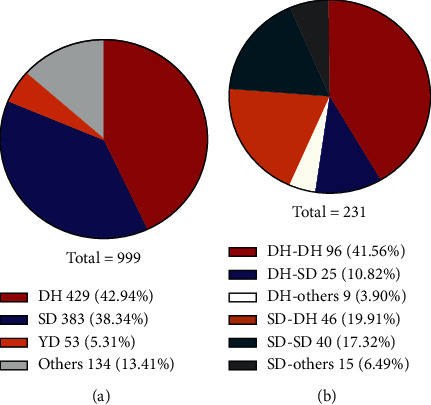
The syndrome distribution in 999 CHB patients and the syndrome conversion after 3-month follow-up. (a) Distribution of TCM syndrome in 999 CHB cases. (b) Conversion of syndrome types in 231 CHB patients after 3-month follow-up.

Among the 999 CHB patients, the proportion of DH syndrome was the highest (%), followed by the SD syndrome (%) (Figure 2(a)). We chose CHB patients with DH syndrome (*n* = 429) or SD syndrome (*n* = 383) to analyze the clinical indexes differences. The ALT, AST, TBA, and PT values of the DH syndrome were significantly higher than those of the SD syndrome (*P*=0.048, *P*=0.024, *P*=0.006, *P*=0.002, respectively). The age and AFP values of patients with DH syndrome were slightly higher than those of patients with SD syndrome, but there was no statistical difference (Table 1). To assess the effect of drugs on differences in liver function, we compared the clinical medications of the two groups of patients. There was no difference in the composition of antiviral drugs, Chinese herbal medicine, and antifibrosis proprietary Chinese medicine between DH syndrome and SD syndrome. However, the proportion of immunomodulators in the DH syndrome group was significantly higher than that in the SD syndrome group (*P* < 0.05). And the proportion of hepatoprotective drugs and drugs that promote bile excretion of the DH syndrome was slightly higher than that in the SD syndrome (*P* < 0.1, Table 1).

### 3.2. Comparison of Clinical Indexes and Drug Use among the Four Main TCM Syndrome Conversion Groups in CHB Patients after 3-Month Follow-Up

Among the 232 CHB patients who were followed up after 3 months, the composition ratio of syndrome changes after 3 months is shown in Figure 1(b). Among the 130 patients diagnosed with DH syndrome for the first time, 96 (73.84%) patients maintained the DH syndrome unchanged (group A), and 25 (19.23%) patients changed from DH syndrome to SD syndrome after 3 months (group B). Among the 101 patients diagnosed with SD syndrome at the first time point, 46 (45.54%) patients changed to DH syndrome after 3 months of follow-up (group C), and 40 (39.60%) patients maintained the SD syndrome unchanged (group D).

We compared the dynamic differences in clinical indicators between patients with syndrome changes and those with unchanged syndromes. As seen in Table 2, there was no significant difference in gender, age, and medication between patients in groups C and D. Compared with group C, the improvement of TBIL, AST, and ALB in group D was more significant (*P* < 0.05), and the improvement trend of ALT, APRI, and FIB-4 in group D was slightly better than that in group C (*P* < 0.1). The results indicate that CHB patients with SD syndrome who converted into DH syndrome have a worse clinical recovery of liver function than those who maintained SD syndrome, which is consistent with the trend of liver damage in the first cross-sectional observation. Although no significant difference was found in the clinical indicator comparation between group A and group B, the CHB patients who have maintained DH syndrome had a worse liver function trend than those of SD syndrome patients. In terms of medication, the untreated patient in group A was significantly higher than that in group B, and the proportion of taking Chinese herbal medicine in group A was significantly lower than that in group B. An additional file shows this in more detail (see Additional file 1).

### 3.3. Analysis of the Factors Related to the Improvement of ALT Level in CHB Patients

We used the logistic regression model to analyze whether TCM syndrome is a variable closely related to a different recovery of serum ALT. Since the serum ALT level of most patients in the follow-up after 3 months was lower than the first time point, we used whether the ALT decreased more than 30% from the baseline level during the follow-up after 3 months as the dependent variable. To exclude the interference of the syndrome conversion, CHB patients in group A or group D whose serum ALT level was abnormal (more than 50U/L) at baseline were included in the model (including 49 patients in group A and 24 patients in group D). Simple logistic regression analysis was used to include syndrome grouping and medication information as independent variables. DH syndrome is a significant negative factor (OR [95% CI]: 4.854 [1.149–20.501], *P*=0.032). Then, we used the multiple logistic regression model, which included age, gender, and HBV-DNA value as covariates; the adjusted OR value of DH syndrome was 4.936 (*P*=0.032). These results indicate that DH syndrome is a negative factor for reducing the serum ALT level in CHB patients (Table 3).

## 4. Discussion

In this study, we analyzed clinical data of 999 CHB patients with TCM syndrome records. Among these patients, DH syndrome and SD syndrome were the two most common syndromes, accounting for 81.72% of the total collected CHB patients, which was consistent with previous research reports [10, 19]. In our study, the ALT and AST levels of patients with DH syndrome were significantly higher than those of the SD group (*P*=0.048, *P*=0.024, respectively), indicating that the liver damage of CHB patients with DH syndrome was higher than that of SD syndrome, which is consistent with the results of previous reports that CHB patients with DH syndrome have higher levels of ALT or AST [11–14]. Besides, some studies have pointed out that the ALT and AST levels of autoimmune hepatitis patients with DH syndrome were significantly higher than those of SD syndrome [20]; patients of NAFLD with DH syndrome also had a significantly higher ALT level than that of other syndrome types [21]. Therefore, we speculate that patients with DH syndrome have a higher level of liver damage which is a common manifestation of chronic liver diseases.

Because HBV is a noncytopathic virus, liver injury is mediated by the immune-mediated cytotoxicity in chronic infection. Therapeutic control of HBV-DNA and ALT in CHB is associated with decreased NK cell killing of HBV-positive hepatocytes and HBV-specific T cells, and increased antiviral T-cell response [22]. In dynamic follow-up, we hope to observe whether patients with different syndromes of CHB also have differences in drug response and disease outcomes. Due to drug intervention, the level of ALT and AST was decreased in varying degrees in most of the follow-up dynamic observation patients, but the improvement trend of TBIL, ALT, and AST of CHB patients with sustained SD syndrome was significantly better than those whose TCM syndrome changed from SD to DH (*P*=0.048, *p*=0.061, *p*=0.033, respectively). Similarly, patients with CHB DH syndrome converted to SD syndrome had a better recovery trend of liver damage than patients with persistent DH syndrome but without statistical difference, which might be related to the imbalanced number of cases between the two groups. As seen in Supplementary Table 1, compared with group A, the difference of ALT in group B was slightly greater (A vs B, −6(−28.75, 6) vs −16(−52.5, −0.5), *P*=0.291). The above results suggest that patients with CHB DH syndrome have potentially more sustained liver function damage than those with SD syndrome.

Persistent liver damage is more likely to be accompanied with HBV-related cirrhosis and hepatocellular carcinoma. In this study, the AFP level of patients with DH syndrome had a higher trend than that of the SD syndrome (Table 1, *P*=0.065). In the dynamic observation, the improvement trend of FIB-4 and APRI scores of patients with sustained SD syndrome showed also a better trend than patients with TCM syndrome converted from SD to DH (Table 2, *P*=0.09 and *P*=0.052, respectively), and there was no difference in the usage of antifibrosis drugs between the two groups. Studies also pointed out that CHB patients with DH syndrome were more likely to be complicated with hepatitis B cirrhosis and liver cancer. The hepatitis B cirrhosis patients with DH syndrome are distributed in Child–Pugh grades A, B, and C, but are mainly in the B and C grades and more prone to having complications, such as upper gastrointestinal hemorrhage and hepatic encephalopathy [23]. The DH syndrome (accounting for 30.97%) also was the most common type in the III stage of liver cancer [24]. Combined with our results, it is suggested that patients with CHB DH syndrome are associated with higher liver damage and potentially higher risk of liver fibrosis and hepatocellular carcinoma.

Medication also has a great effect on liver function, so we compared the difference in medication between the two groups of patients. In the first part of the cross-sectional study, CHB patients with DH syndrome had significantly higher level of ALT, AST, and TBA, but took more immunomodulators and hepatoprotective drugs than the SD syndrome group (*P*=0.021 and *P*=0.081). In the second part of the dynamic follow-up observation, there was no difference between the medications of the patients in group C and group D, but the rate of taking traditional Chinese herbal medicine in group A was significantly lower than that in group B, and ALT had a poor improvement trend in group A (Additional file 1). Therefore, in order to further clarify the influence of syndromes and medication information on the improvement of ALT, we conducted the logistic analysis to screen for risk factors that affect the improvement of ALT. The regression analysis results showed that the decrease of ALT is related to the syndrome types; DH syndrome is an adverse effect on the reduction of ALT in CHB patients. However, due to the small difference in medication between the two groups, the drugs were not statistically significant in models.

The biological basis of CHB patients with DH syndrome having a higher level of ALT is still unclear; some researches suggest that the expression of inflammatory cytokines of CHB patients with DH syndrome is relatively higher and the immune response is hyperactive. The CHB patients with DH syndrome had a significantly higher level of serum MIP-1*α* than that of patients with liver-kidney yin deficiency syndrome [25]. In addition, the A allele proportion of TNF-*α*-308 gene in patients with DH syndrome of hepatitis B cirrhosis is significantly higher than that of non-DH syndrome [26]. Yinchenhao Decoction (YCHD) is a classic prescription for treating jaundice with DH syndrome. Network pharmacological studies have shown that YCHD can treat chronic liver diseases through functional modules such as immune response, inflammation, energy metabolism, and so on. However, compared with Huangqi decoction for qi deficiency syndrome, YCHD has unique functional modules related leptin-induced complement pathway and leukocyte migration across the endothelium, which may correlate with the anti-inflammatory effects [27].

## 5. Limitations

There are still some problems in this study. For example, this is a cross section study with a low level of evidence. The number of research cases is not enough, especially in the dynamic analysis. Therefore, we need to constantly expand the sample size to verify and modify the results.

## 6. Conclusion

In this study, we explored the clinical and medication differences of CHB patients with two common TCM. Based on the analysis of combined medication in the real world, we found that CHB patients with DH syndrome have potentially more serious and sustained liver function damage than those with SD syndrome. The DH syndrome is a negative factor for reducing serum ALT level in CHB patients. This study provides a reference for the personalized management and treatment of chronic hepatitis B patients from the perspective of TCM syndromes.

## Figures and Tables

**Table 1:** Baseline analysis of 812 CHB patients with DH syndrome and the SD syndrome.

	DH (*n* = 429)	SD (*n* = 383)	Statistics	*P*
Gender (male, %)	326 (75.99%)	213 (55.61%)	0.889^a^	0.346
Age (years)	44.44 (37.1, 54.61)	44.06 (36.22, 51.38)	−1.887	0.059
*Treatment*				
Antiviral therapy	259 (60.37%)	231 (60.31%)	0.000^a^	0.986
Recent anti-virus^*∗*^	60 (23.34%)	51 (22.76%)	0.023^a^	0.881
Antivirus duration (days)	325 (96, 758.5)	370.5 (101, 1074.5)	−1.378	0.168
Hepatoprotective drugs	258 (60.13%)	253 (66.05%)	3.037	0.081
Immunomodulator	15 (3.49%)	4 (1.04%)	5.324^a^	**0.021**
Drugs that promote bile excretion	46 (10.72%)	28 (7.31%)	2.844^a^	0.092
Chinese herbal medicine	108 (25.65%)	99 (25.84%)	0.048^a^	0.826
Anti-fibrosis proprietary Chinese medicine	18 (4.19%)	21 (5.48%)	0.733^a^	0.392
Untreated	81 (18.88%)	70 (18.27%)	0.049^a^	0.825
*Liver function value*				
TBIL (*μ*mol/L)	16.6 (12.5, 22.47)	15.7 (12.47, 21.08)	−1.119	0.263
DBIL (*μ*mol/L)	4.4 (3.3, 6.7)	4.4 (3.03, 6.3)	−0.994	0.32
ALT (U/L)	44 (26, 92.5)	40.8 (23.3, 79.75)	−1.977	**0.048**
AST (U/L)	36 (26.02, 60.75)	33 (24, 56)	−2.25	**0.024**
GGT (U/L)	29.85 (18, 65.5)	28 (17.77, 55.25)	−1.352	0.176
ALP (U/L)	79 (64, 100)	75.15 (63, 96)	−1.617	0.106
ALB (g/L)	45.5 (43, 47.8)	45.4 (42.62, 48.1)	−0.315	0.753
Pre-Alb (g/L)	241 (178, 290)	237.35 (180, 288.5)	−0.043	0.966
TBA (*μ*mol/L)	7.6 (3.9, 14)	5.9 (2.9, 12.8)	−2.749	**0.006**
*Hepatitis B virus*				
HBeAg positive (%)	227 (53.53%)	186 (49.33%)	1.410^a^	0.235
HBV-DNA (IU/mL)	13020 (0, 2270000)	7181 (0, 2115000)	−1.245	0.213
*Liver fibrosis value*				
APRI	0.48 (0.31, 0.91)	0.46 (0.27, 0.80)	−1.429	0.153
FIB-4	1.63 (1.07, 2.77)	1.52 (1.01, 2.33)	−1.603	0.109
*Other biomarkers*				
PT (S)	13.2 (12.2, 14.4)	12.7 (12, 13.9)	−3.054	**0.002**
AFP (ng/mL)	3.67 (2.6, 6.5)	3.45 (2.36, 5.84)	−1.845	0.065
CD4/CD8	1.33 (1.02, 1.76)	1.26 (0.89, 1.73)	−1.641	0.101
*Lipids, Blood glucose*				
FBG (mmol/L)	5.18 (4.85, 5.47)	5.14 (4.85, 5.5)	−0.601	0.548
TC (mmol/L)	4.59 (3.83, 5.2)	4.58 (3.96, 5.22)	−0.891	0.373
TG (mmol/L)	1.1 (0.82, 1.49)	1.07 (0.79, 1.52)	−0.281	0.779
HDL-C (mmol/L)	1.18 (1, 1.415)	1.2 (1.01, 1.43)	−0.666	0.506
LDL-C (mmol/L)	2.54 (2.02, 2.94)	2.46 (2.11, 3.05)	−0.611	0.541
*Blood routine test*				
WBC (10^9^/L)	5.01 (4.26, 6.01)	5 (4.13, 6.04)	−0.259	0.796
LY (10^9^/L)	1.64 (1.28, 2)	1.68 (1.32, 2.04)	−0.656	0.512
MONO (10^9^/L)	0.31 (0.24, 0.4)	0.32 (0.26, 0.41)	−0.769	0.442
NEUT (10^9^/L)	2.89 (2.25, 3.6)	2.8 (2.16, 3.65)	−0.749	0.454
PLT (10^9^/L)	159 (124, 202.5)	159 (122, 198)	−0.151	0.88
*Renal function value*				
BUN (mmol/L)	4.48 (3.61, 5.2)	4.37 (3.6, 5.11)	−0.718	0.473
Cr (*μ*mol/L)	70 (59.78, 80)	68 (60, 78)	−1.253	0.21
UA (*μ*mol/L)	300 (256.5, 354)	303.5 (244, 352)	−0.37	0.712

^*∗*^The time interval between the start date of antiviral treatment and the enrollment date ≤90 days. APRI = (AST (U/L)/upper limit of normal value (U/L))/PLT (×10^9^/L) ×100; FIB-4 = age (year) ×AST (U/L)/(PLT(×10^9^/L) × ALT(U/L)^1/2^).

**Table 2:** Comparison of clinical index difference and medication between group C and group D.

	Group C (SD to DH) *n* = 46	Group D (sustained SD) *n* = 40	Statistics	*P*
Gender (male, %)	32 (69.56%)	32 (80%)	1.224^a^	0.269
Age (years)	41.64 (36.54, 51.49)	48.48 (37.64, 51.98)	−1.056	0.291

*Treatment*				
Antiviral therapy	26 (56.52%)	28 (70%)	1.664^a^	0.197
Recent antivirus	10 (21.73%)	9 (22.5%)	0.007^a^	0.932
Antivirus duration (days)	116 (53.5, 515)	173 (53.5, 416)	−0.090	0.928
Hepatoprotective drugs	18 (39.13%)	19 (47.5%)	0.611^a^	0.434
Immunomodulator	0 (0%)	0 (0%)	—	—
Drugs that promote bile excretion	4 (8.69%)	3 (7.5%)	0.041^a^	0.579
Chinese herbal medicine	17 (36.95%)	15 (37.5%)	0.003^a^	0.959
Antifibrosis proprietary Chinese medicine	1 (2.2%)	3 (7.5%)	1.369^a^	0.257
Untreated	4 (8.69%)	0 (0%)	3.648^a^	0.077

*Liver function value*				
TBIL (*μ*mol/L)	0.04 (−1.87, 3.14)	−1.6 (−6.65, 2.22)	−1.979	**0.048**
DBIL (*μ*mol/L)	0 (−0.7, 1)	0 (−1.72, 1.12)	−0.792	0.428
ALT (U/L)	−11 (−34, 5.75)	−19 (−96, −6.25)	−1.875	0.061
AST (U/L)	−2 (−15.25, 9.75)	−12.5 (−69, 1)	−2.130	**0.033**
GGT (U/L)	−1 (−23, 3)	−8.5 (−51, 3)	−1.178	0.239
ALP (U/L)	0.5 (−11.25, 8.25)	−3 (−13, 8.75)	−0.723	0.469
ALB (g/L)	1 (−2.32, 2.82)	2.19 (0.05, 5.15)	−2.156	**0.031**
Pre-Alb (g/L)	0 (−112, 64.5)	19 (−116.5, 70)	−0.004	0.996
TBA (*μ*mol/L)	−2.3 (−5.05, 0.02)	−2.75 (−15.07, −0.27)	−1.130	0.258

*Hepatitis B virus*				
HBeAg (S/CO)	−0.075 (−155.5925, 0.775)	−3.135 (−90.502, 0.385)	0.444^a^	0.505
HBV-DNA (IU/mL)	−35280 (−2775275, 643.75)	−25080.5 (−5387429.25, 0)	−0.320	0.749

*Liver fibrosis value*				
FIB-4	0.04 (−0.21, 0.36)	−0.22 (−0.86, 0.42)	−1.697	0.09
APRI	−0.02 (−0.1826, 0.069)	−0.18 (−0.83, 0.02)	−1.939	0.052

*Other biomarkers*				
PT (S)	−0.24 (−0.82, 0.52)	0 (−0.75, 0.67)	−0.671	0.502
AFP (ng/mL)	−1.13 (−3.50, 0.05)	−0.97 (−3.18, −0.05)	−0.089	0.929
CD4/CD8	0.06 (−0.18, 0.37)	0.07 (−0.13, 0.31)	−0.320	0.749

*Lipids, blood glucose*				
FBG (mmol/L)	−0.09 (−0.51, 0.06)	0.24 (−0.03, 0.50)	−3.355	**0.001**
TC (mmol/L)	−0.27 (−0.73, 0.13)	−0.32 (−1.30, 0.2)	−0.377	0.706
TG (mmol/L)	−0.03 (−0.26, 0.13)	−0.08 (−0.57, 0.19)	−0.610	0.542
HDL-C (mmol/L)	−0.16 ± 0.33	−0.09 ± 0.51	−0.818	0.416
LDL-C (mmol/L)	−0.10 ± 0.69	−0.31 ± 1.04	1.103	0.273

*Blood routine test*				
WBC (10^9^/L)	0.14 ± 1.19	0.39 ± 1.23	−0.964	0.338
LY (10^9^/L)	0.04 ± 0.40	0.07 ± 0.53	−0.306	0.76
MONO (10^9^/L)	0.01 (−0.10, 0.10)	−0.02 (−0.16, 0.08)	−0.737	0.461
NEUT (10^9^/L)	0.11 (−0.4, 0.71)	0.55 (−0.19, 1.23)	−1.367	0.172
PLT (10^9^/L)	−4.59 ± 27.25	5.63 ± 31.57	−1.610	0.111

**Table 3:** Analysis of factors related to the decrease of ALT.

	ALT decreased by more than 30%			
	Crude OR^*∗*^ [95% CI]	*P*	Adjusted OR^†^ [95% CI]	*P*
DH syndrome	4.854 (1.149, 20.501)	**0.032**	4.936(1.144, 21.307)	**0.032**
*Medication*				
Antiviral therapy	1.638 (0.329, 8.143)	0.547	1.638 (0.329, 8.143)	0.547
Hepatoprotective drugs	0.561 (0.136, 2.313)	0.424	0.542 (0.123, 2.382)	0.418
Drugs that promote bile excretion	1.371 (0.303, 6.206)	0.682	1.517 (0.235, 9.777)	0.661
Chinese herbal medicine	0.292 (0.05, 1.728)	0.175	0.26 (0.04, 1.684)	0.158
Untreated	0.883 (0.118, 6.604)	0.904	1.025 (0.124, 8.453)	0.982

Analysis of factors related to the decrease of ALT in patients with ALT >50 U/L at baseline of group A&D. ^*∗*^Crude OR estimated by the simple logistic regression model. ^†^Adjusted OR estimated by multiple logistic regression models including age, gender, and HBV-DNA value as covariates.

## Data Availability

The data used to support the findings of this study are available from the corresponding author upon request.

## Supplementary Materials

Supplementary Materials Additional file 1 (Supplementary Table 1. Comparison of clinical index difference and medication between group A and group B). Additional file 2 (Diagnostic criteria for damp-heat and spleen deficiency syndrome differentiation of CHB patients).
