# Supplementary material for: Comparative Analysis of Clinical and Medication Information between Chronic Hepatitis B Patients with Damp Heat Syndrome and Spleen Deficiency Syndrome
Source: Evid Based Complement Alternat Med. 2020 Dec 28;2020:8846637. doi: 10.1155/2020/8846637 (PMC7781698; doi:10.1155/2020/8846637)
Supplement: Supplementary Materials — Additional file 1 (Supplementary Table 1. Comparison of clinical index difference and medication between group A and group B). Additional file 2 (Diagnostic criteria for damp-heat and spleen deficiency syndrome differentiation of CHB patients). [file 8846637.f1.zip › 8846637.f1/Additional file 1 (1).docx]

**Supplementary Table 1** Comparison of clinical index difference and medication between group A and group B.

| **Item** | **Group A**  (Sustained DH)  ***n* =96** | **Group B**  (DH to SD）  ***n* =25** | | **Statistics** | **P-value** |
| --- | --- | --- | --- | --- | --- |
| Gender(Male%) | 77(80.20%) | 22(88%) | | 0.809^a^ | 0.368 |
| Age(years) | 43.4(37.48,53.4) | 40.61(36.56,48.52) | | -0.813 | 0.416 |
| **Treatment** | | | | | |
| Antiviral therapy | 65(67.70%) | 16(64%) | | 0.123^a^ | 0.726 |
| Recent anti-virus | 21(32.3%) | 6(37.5%) | | 0.052^a^ | 0.820 |
| Antivirus duration (days) | 215(71.5,440) | 108(26.5,411) | | -0.508 | 0.612 |
| Hepatoprotective drugs | 60(62.5%) | 13(52%) | | 0.914^a^ | 0.339 |
| Immunomodulator | 5(5.20%) | 0(0%) | | 1.358^a^ | 0.244 |
| Drugs that promote bile excretion | 12(12.5%) | 3(12%) | | 0.005^a^ | 0.946 |
| Chinese herbal medicine | 14(14.58%) | 11(44%) | | 10.471^a^ | **0.001** |
| Anti-fibrosis proprietary Chinese medicine | 0(0%) | 0(0%) | | - | **-** |
| Untreated | 15(15.625%) | 0(0%) | | 4.459^a^ | **0.024** |
| **Liver function value** | | | |  |  |
| TBIL(umol/L) | -0.75(-5.02,3） | -0.19(-3.55,2.65） | | -0.240 | 0.810 |
| DBIL(umol/L) | 0.05(-1.7,1.2） | 0.5(-1.4,1.1） | | -0.653 | 0.514 |
| ALT(U/L) | -6(-28.75,6） | -16(-52.5,-0.5） | | -1.057 | 0.291 |
| AST(U/L) | -5(-15.75,3） | -7(-30,-1.5） | | -1.322 | 0.186 |
| GGT(U/L) | -1(-26,5.75） | -4(-26.5,3.5） | | -0.387 | 0.698 |
| ALP(U/L) | -1(-12,9.75） | -5(-7,4.5） | | -0.054 | 0.957 |
| ALB(g/L) | 0.75(-1.29,3.07） | -0.29(-1.79,1.39） | | -1.476 | 0.140 |
| Pre-Alb(g/L) | 39(-16,94） | 40(-6,85） | | -0.071 | 0.943 |
| TBA(umol/L) | -3.4(-14.49,0.2） | -2.2(-7.95,-0.05） | | -0.675 | 0.499 |
| **Hepatitis B virus** | | | |  |  |
| HBeAg(S/CO) | -0.01(-65.00,42.01） | | -0.08(-149.07,9.595） | 0.780 | 0.411 |
| HBV-DNA(IU/mL) | -17930.5(-2497200,1627.25） | -11770(-1846926.5,3121000） | | -1.319 | 0.187 |
| **Liver fibrosis value** | | | |  |  |
| FIB-4 | -0.07(-0.33,0.32） | -0.17(-0.84,0.05） | | -1.383 | 0.167 |
| APRI | -0.05(-0.23,0.05） | -0.09(-0.42,-0.004） | | -1.370 | 0.171 |
| **Other biomarkers** | | | |  |  |
| PT(S) | 0.10(-0.47,0.77） | 0.19(-0.55,0.80） | | -0.176 | 0.860 |
| AFP(ng/mL) | -0.43(-2.2,0.28） | -0.48(-1.43,0.19） | | -0.259 | 0.796 |
| CD4/CD8 | 0.01(-0.22,0.27） | -0.008(-0.28,0.30） | | -0.070 | 0.944 |
| **Lipids, Blood glucose** | | | |  |  |
| FBG(mmol/L) | -0.06(-0.3,0.27） | -0.19(-0.4,0.05） | | -1.076 | 0.282 |
| TC(mmol/L) | -0.37±0.91 | -0.40±1.03 | | 0.148 | 0.882 |
| TG(mmol/L) | -0.15(-0.38,0.18） | -0.07(-0.37,0.21） | | -0.698 | 0.485 |
| HDL-C(mmol/L) | -0.21±0.36 | -0.10±0.36 | | -1.345 | 0.181 |
| LDL-C(mmol/L) | -0.14(-0.54,0.28） | 0.03(-0.81,0.21） | | -0.266 | 0.790 |
| **Blood routine test** | | | |  |  |
| WBC(10^9^/L) | 0.005(-0.71,0.75） | -0.08(-0.57,0.57） | | -0.198 | 0.843 |
| LY(10^9^/L) | 0.01(-0.20,0.25） | 0(-0.32,0.17） | | -0.467 | 0.640 |
| MONO(10^9^/L) | -0.02(-0.09,0.02） | 0(-0.07,0.08） | | -1.144 | 0.253 |
| NEUT(10^9^/L) | 0.09(-0.44,0.71） | 0.03(-0.36,0.6） | | -0.032 | 0.974 |
| PLT(10^9^/L) | -3(-23,18） | -2(-14,10.5） | | -0.291 | 0.771 |

*The difference is equal to the post-follow-up value minus pre-follow-up value.*
