# Supplementary material for: Comparative Analysis of Clinical and Medication Information between Chronic Hepatitis B Patients with Damp Heat Syndrome and Spleen Deficiency Syndrome
Source: Evid Based Complement Alternat Med. 2020 Dec 28;2020:8846637. doi: 10.1155/2020/8846637 (PMC7781698; doi:10.1155/2020/8846637)
Supplement: Supplementary Materials — Additional file 1 (Supplementary Table 1. Comparison of clinical index difference and medication between group A and group B). Additional file 2 (Diagnostic criteria for damp-heat and spleen deficiency syndrome differentiation of CHB patients). [file 8846637.f1.zip › 8846637.f1/Additional file 2.docx]

**Diagnostic criteria for damp-heat and spleen deficiency syndrome differentiation of CHB patients**

**Damp-heat syndrome:** hypochondrium and stomach cavity is of distention and fullness, nausea and aversive greasy, anorexia, **the skin and eyes are yellow-stained with bright color, the urine is yellow,** stickiness **and** bitter taste **in the mouth, the stool is sticky and greasy** accompanying with an offensive odour, or the stool is dry first and then loose, thirsty and want to drink, or thirsty but drink less, limbs **are sleepy and heavy,** burnout and fatigue, yellow and greasy tongue coating, pulse manifestation is of string slippery or number of strings.

Primary symptoms: (1) the skin and eyes are yellow-stained with bright color. (2) tongue coating is yellow and greasy.

Secondary symptoms: (1) nausea, aversive greasy, anorexia. (2) hypochondrium and stomach cavity is of distention and fullness. (3) the urine is yellow.

Those belong to the damp-heat syndrome who have the primary symptoms (1) and (2) listed above, or who have the primary symptoms (2) and any two of the three secondary symptoms, or who have the primary symptoms (1) and secondary symptoms (1) and (2).

**Spleen deficiency syndrome:** distending pain in hypochondrium, oppression in chest and sighing, depression, quick temper, eating less, tastelessness and loss of appetite, gastric stuffiness and abdominal distention, aggravation in the afternoon, shortage of qi and be tired of talking, fatigue, complexion is of wilted yellow, sloppy diarrhea or diarrhea with undigested food, and which will be aggravated by eating cold, greasy and indigestible food, pale tongue with tooth prints, white tongue coating, sinking string pulse.

Primary symptoms: (1) distending pain in hypochondrium. (2) abdominal distention and sloppy diarrhea.

Secondary symptoms: (1) depression and boredom. (2) fatigue and lack of strength. (3) pale tongue with tooth prints.

Those belong to the spleen deficiency syndrome who have the primary symptoms (1) and (2) listed above, or who have the primary symptoms (1) and secondary symptoms (2) and (3), or who have the primary symptoms (2) and secondary symptoms (1).
